# The economic burden of myopia diagnosis and treatment: A cross-sectional cost-of-illness study in Yantai, China

**DOI:** 10.1097/MD.0000000000051282

**Published:** 2026-10-23

**Authors:** Yuelin Sun, Xiaosong Cheng, Cong Sun, Naibao Hu, Xiaoxiang Jin, Xiaoyue Wang, Fei Wang, Guiqiang Wang

**Affiliations:** a Department of School Health, Yantai Center for Disease Control and Prevention, Yantai, Shandong, China; b Department of School Health, Zhifu District Center for Disease Control and Prevention, Yantai, Shandong, China; c School of Public Health, Shandong Medical and Pharmaceutical University, Yantai, Shandong, China.

**Keywords:** cost-of-illness, cross-sectional study, economic burden, myopia

## Abstract

**Background:**

Myopia has become a major global public health and economic issue. Population-based cost-of-illness evidence from prefecture-level cities in China remains limited. This study aimed to quantify the direct economic burden of myopia diagnosis and treatment among individuals aged 6 to 55 years in Yantai, China, and to identify factors associated with per-capita direct myopia expenditure.

**Methods:**

A cross-sectional cost-of-illness study was conducted between September and November 2021 in Yantai. A multistage sampling strategy was used to recruit students and their parents from primary schools, middle schools, high schools/vocational schools, and universities. Questionnaire data on demographic characteristics, myopia status, correction methods, healthcare utilization, and costs were collected. A gamma generalized linear model with a log link was used to examine factors associated with total myopia expenditure.

**Results:**

A total of 30,024 participants were included in the analysis (from 30,378 who participated). The prevalence of myopia was 36.81% (n = 11,053), with a higher rate in females (38.70%) than in males (34.95%). Spectacles were the most common correction method (94.93%). The median annual direct cost per myopic patient was 300.00 Chinese Yuan (CNY), accounting for 60.00% of total medical expenses. Children aged 6 to 10 years had the highest annual direct cost (829.50 CNY). In the gamma generalized linear model, among myopic patients with positive expenditures, age and educational level were significant predictors of the direct economic burden. For each 1-year decrease in age, the annual per capita direct cost increased by approximately 38.53 CNY (*P* < .01). Compared with those with postgraduate education or above, patients with primary school education or below and those with junior high school education incurred an additional 1154.61 and 226.67 CNY, respectively (*P* < .01), whereas gender had no significant effect (*P* = .39).

**Conclusion:**

Myopia imposes a substantial economic burden in Yantai, especially among younger individuals, those with early-onset myopia, individuals with lower educational attainment, and those using expensive correction methods.

## 1. Introduction

Myopia is a complex eye disease resulting from the interaction between genetic and environmental factors. Myopia has become a significant public health concern.^[1]^ Studies have indicated that nearly one-third of the world’s population suffers from myopia. By 2050, approximately half of the world’s population is expected to be myopic, with approximately 10% suffering from high myopia.^[2]^ A study conducted in East and Southeast Asia showed that myopia increased sharply over the past 60 years, with 80% to 90% of school-age children affected.^[3]^ In 2020, the myopia rate among Chinese children and adolescents was 52.7%, which is significantly higher than that in the United States, Australia, and other countries. Current science and technology allow myopia to be corrected through spectacles, contact lenses, or surgery. However, a cure remains elusive, and further progression remains a risk. If effective measures are not taken to control the development of myopia, it may lead to a series of eye complications, including age-related macular degeneration, glaucoma, and retinal detachment, ultimately resulting in permanent vision loss and reduced quality of life.^[4]^ This not only impacts patients’ lives but also significantly increases medical costs, imposing a substantial economic burden on patients and their families.^[5]^ In 2018, costs worldwide linked to myopia-related blindness – including direct healthcare expenses, lost productivity, and social security costs – amounted to >US$670 billion. Projections indicate that these costs will reach US$1.7 trillion by 2050.^[6]^

The increasing prevalence of myopia in China poses significant challenges to public health. Given China’s large population, it is projected that approximately 180.4 million children aged 7 to 18 will develop myopia by 2030.^[7]^ With an aging population, the economic burden of myopia-related costs (including diagnosis, spectacle correction, surgical treatment, regular follow-up, absenteeism, and complication treatment) significantly affects both individuals and society, as well as the future health quality of the nation. Systematic research on the economic burden of myopia-related diseases is paramount. On the 1 hand, understanding the negative impact of myopia on China’s economy and society is crucial. On the other hand, this research also provides a basis for local health resource planning and for further research in other settings.

In this study, self-assessment questionnaires were distributed among students of primary school, junior high school, high school, vocational high school, and university, as well as parent questionnaires for primary school to vocational high school students. These questionnaires were employed to assess the direct and indirect economic burden of myopia patients aged 6 to 55 in Yantai, providing a theoretical basis for the prevention and control of myopia and allocation of health resources.

## 2. Methods

### 2.1. Research time and place

This study was conducted between September and November 2021. Based on regional characteristics, Yantai is a prefecture-level city in Shandong province, located northeast of the Shandong Peninsula in eastern China. The terrain of this area, characterized by low hills and ridges, supports a temperate monsoon climate. By the end of 2022, the total land area was 13,930.1 km^2^. According to statistics from the Yantai Municipal Bureau, as of the end of 2021, the city had a permanent population of approximately 7.0828 million. In 2021, Yantai’s gross domestic product reached 871.175 billion Chinese Yuan (CNY). The average annual salary for urban employees outside the private sector was 94,524 CNY, while the per capita disposable income for all residents was 42,629 CNY. Urban and rural residents had per capita disposable incomes of 53,169 and 24,574 CNY, respectively.

### 2.2. Research population

This study was designed as a cross-sectional cost-of-illness analysis conducted from a societal perspective, it does not evaluate the cost-effectiveness of alternative interventions. Considering the possible influencing factors for the disease’s economic burden, the study population was myopic individuals aged 6 to 55 in Yantai city in 2021, including urban populations from 7 districts and rural populations from 7 counties. Seven schools were chosen in each urban district: 2 primary schools, 2 middle schools, 1 senior high school, 1 secondary vocational school, and 1 university. Five schools were selected in each county-level city: 2 primary schools, 2 middle schools, and 1 senior high school. Two or more classes were randomly sampled from each grade of the selected schools, as well as from grades 1 to 3 of the university. Students from these classes participated in the monitoring, ensuring that at least 80 students were included per grade. Students from primary school to high school and their parents were asked whether they had signed the written informed consent form under the agreement, and college students were also investigated. All students from primary school to high school and their parents, and the university students were invited if they had signed the written informed consent.

Referring to the Screening Standard for Myopia in Children and Adolescents issued by the National Health Commission of the People’s Republic of China,^[8]^ myopia in this study was defined as the condition that the monocular uncorrected distance visual acuity is <5.0, and the spherical equivalent refractive error measured under non-cycloplegic or cycloplegic refraction is ≤−0.50 diopters (D). The clinical criterion for diagnosing myopia in adults is a spherical equivalent of ≤−0.50 D measured after cycloplegia. The survey contents included myopia-related information on the subjects, including general information, myopia status, myopia management strategies, myopia-related expenses, and myopia correction frequency. A total of 30,378 individuals in Yantai city participated in this study, of which 30,064 were eligible to complete the questionnaire, with a response rate of 98.97%. Among them, 30,024 participants aged 6 to 55 were included in the final analysis (effective rate 99.87%).

### 2.3. Questionnaire and its reliability

The questionnaire was developed to assess the financial burden of myopia among survey participants, categorized into 2 groups based on age demographics: 1 for primary and junior high school students, and another for parents and university students. Both questionnaires encompassed myopia-related information, corrective measures (including spectacles and myopia surgery), diagnosis and treatment (including hospital and outpatient visits, visit frequency, accompanying persons, and associated costs), vision testing, and myopia prevention and control measures. The questionnaire for parents and university students also incorporated basic family information, including the number of family members and income.

### 2.4. Quality control

To ensure the quality of the survey, the researchers in each city underwent uniform training based on a self-prepared manual. Cost data are retrospective and self-reported. To improve data accuracy, we conducted uniform training of investigators, provided on-site explanations, held parent meetings, and established real-time question-and-answer groups. Before completing the survey, the researchers were required to explain the study’s objectives, methodologies, and instructions for completion. For university students, researchers distributed and explained the survey on-site, providing students with the opportunity to seek clarification if needed. Parents completed the survey on behalf of their primary, junior high, and senior high school students. Researchers organized parent meetings to elucidate the purpose of the study and established online groups for real-time Q&A sessions to address any concerns. If parents were unable to participate, other guardians were invited to complete the survey.

The researchers randomly sampled 10 questionnaires from each class for quality assurance. If >10% of the items were missing, or if the responses were illogical or unrealistic, the questionnaire was deemed unqualified and returned to the students for recompletion. If more than 2 questionnaires in a class were found to be unqualified, an additional 10 were selected for further inspection. If >2 unqualified questionnaires were identified in a class, the entire class was required to restart the process.

Data collected from the questionnaires were entered into EpiData version 3.1 (EpiData Association), where automatic checks were performed during data entry. Following data entry, quality was evaluated by comparing the entered variables with the original questionnaire responses. Approximately 10% of the questionnaires were randomly sampled to evaluate the quality of the data entry.

### 2.5. Cost calculation

#### 2.5.1. Direct and indirect economic burden of myopia

We quantified the direct and indirect economic burden of myopia for each participant. The direct economic burden primarily encompasses expenses related to ophthalmic examinations, corrective lenses (including spectacles, orthokeratology/rigid gas permeable [OK/RGP] lenses, and contact lenses), and myopic surgery. The variability in the cost of ophthalmic examinations can be attributed to differences in the type of examination and provider. Corrective lenses represent the predominant expenditure associated with myopia correction. Furthermore, myopia-related surgical interventions are often costly, and their coverage by insurance schemes is inconsistent, thereby imposing substantial out-of-pocket financial burdens on patients. From a societal perspective, the primary funding for myopia-related medical and rehabilitation facilities originates from national budgets and societal contributions.

In this study, the direct economic burden of myopia diagnosis and treatment was defined as the costs incurred by individuals and the healthcare system to diagnose and manage myopia. This includes the expenses associated with purchasing spectacles, OK/RGP lenses, regular contact lenses, and related consumables; the costs of examinations and/or treatments conducted in hospitals or other healthcare institutions; transportation costs for myopia treatment; the expenses related to myopia prevention and control measures; and the costs associated with myopia surgery and related expenses. The indirect economic burden of myopia is defined as the indirect economic impact of myopia on individuals and society, mainly comprising the time lost by patients and caregivers during myopia diagnosis and treatment.

#### 2.5.2. Annual cost per myopic patient

In this study, we calculated the total annual cost of myopia diagnosis and treatment for each patient over the past year. The investigation was conducted between September and November 2021, and the costs were limited to the period before the survey date. The costs refer to the 12 months preceding the survey (September–November 2021), and all costs are expressed in 2021 CNY.

The total annual cost was calculated as follows:


$$\begin{array}{cc} The\;direct\;annual\;cost\;per\;capita\;for\;myopic\;patients\\ & =the\;cost\;of\;purchasing\;spectacles\;and\;\\ & related\;consumables\;in\;the\;previous\;year\\ & +the\;cost\;of\;hospital\;visits\;for\;myopia\;\\ & diagnosis\;and\;treatment\;in\;the\;previous\;year\\ & +the\;cost\;of\;nonhospital\;visits\;in\;the\;previous\;year\\ & +the\;cost\;of\;myopia\;prevention\;and\;control\;in\;the\;previous\;year\\ & +the\;cost\;of\;myopia\text{-}related\;surgeries\;in\;the\;previous\;year. \end{array}$$

The average annual cost of myopia surgery per patient was defined as the total medical cost of myopia surgery divided by the number of patients with myopia who underwent surgery in the previous year.

### 2.6. Statistical analysis

Descriptive statistics were used to analyze the demographic characteristics, myopia status, myopia correction measures, myopia-related expenses, and myopia correction frequency in the study population. The distribution of cost data was examined prior to analysis. Cost data were right-skewed, as indicated by means exceeding medians, and demonstrated substantial variability, with coefficients of variation >1. Accordingly, medians and interquartile ranges were used as the primary measures to describe the typical cost per patient, while means and standard deviations were also reported to facilitate comparability with other cost-of-illness studies. This approach provides a robust description of the central tendency of skewed cost data while preserving comparability with previously published estimates.

The direct economic burdens of myopia were calculated and compared across different age groups, sexes, and educational levels. The association between myopia-related expenses and demographic characteristics was analyzed using the gamma generalized linear model with a log link. All statistical analyses were conducted using R statistical software package, and *P* < .05 was considered statistically significant.

### 2.7. Patient and public involvement

All participants were informed of the study objectives and potential benefits to the public. The results of this study will be submitted to the government to inform the development of myopia-control policies in China.

### 2.8. Ethics approval and informed consent

The study was approved by the Ethics Committee of the Yantai Center for Disease Control and Prevention and adhered to the principles of the Declaration of Helsinki (ethical approval number: YYLLSL202513). Written informed consent was obtained from the parents of primary, middle, and high school students, who signed an agreement with their children. Written informed consent was provided by the university students.

## 3. Results

### 3.1. Myopia research population and prevalence

In total, 30,378 Yantai individuals participated in this study; 30,064 participants completed the questionnaire, resulting in a valid questionnaire rate of 98.97%. Among the eligible respondents, 30,024 participants aged 6 to 55 years were included in the analysis, achieving an effective rate of 99.87%. Of these participants, 15,069 were male (50.19%), and 14,955 were female (49.81%), with an average age of 32 ± 14 years. Among these, 11,053 individuals were diagnosed with myopia, with a prevalence rate of 36.81%. The number of men and women with myopia was 5266 (34.95%) and 5787 (38.70%), respectively. There was a significant difference in the prevalence of myopia between men and women (*P* < .01), with a higher prevalence in women. The age group with the highest prevalence rate of myopia was 16 to 25 years old (78.89%). Conversely, the lowest prevalence rate was found in the 46 to 55 age group (13.21%). Additionally, there was a significant difference in myopia prevalence across the different age groups (*P* < .01) (Table 1).

**Table 1:** Prevalence of myopia in the study population.

Classification		No.	Myopia, no. (%)*	*P* value*
Gender	Male	15,069	5266 (34.95)	<.01
	Female	14,955	5787 (38.70)	
Age (yr)	6–10	1865	612 (32.82)	<.01
	11–15	5189	3465 (66.78)	
	16–25	3491	2754 (78.89)	
	26–35	701	285 (40.66)	
	36–45	14,417	3361 (23.31)	
	46–55	4361	576 (13.21)	
Total		30,024	11,053 (36.81)	

The number and the proportion (%) of the included participants classified by age interval and sex are presented.

* χ^2^ test of myopia prevalence of sample population among different sample populations.

### 3.2. Adoption of different correction methods and costs

Myopia correction measures: Among the 11,053 individuals diagnosed with myopia, 10,493 (94.93%) chose spectacles as their primary correction measure. The median cost was 440.00 CNY. Only 1.95% of individuals opted for OK/RGP lenses, with a median cost of 8000.00 CNY. Additionally, 2.76% of individuals selected regular contact lenses, with an average annual cost of 900 CNY. Another 0.19% of individuals chose other correction measures, with an average annual cost of 880 CNY. Finally, 19 individuals (0.17%) elected not to wear corrective measures. The annual cost was highest for the use of OK/RGP lenses and lowest for the use of spectacles (Table 2).

**Table 2:** Costs of different correction approaches.

Type of vision correction	No. (%)	Mean	SD	Median (*P*25, *P*75)	*P* value*
Spectacles	10,493 (94.93)	548.00	468.04	440.00 (299.90, 630.00)	<.01
OK/RGP lenses	215 (1.95)	8082.83	3761.19	8000.00 (5660.00, 9945.00)	
Regular contact lenses	305 (2.76)	1146.58	928.16	900.00 (480.00,1440.00)	
Other	21 (0.19)	2370.00	2686.28	880.00 (490.00, 5600.00)	

* Mann–Whitney *U* test for comparing costs of different correction approaches across correction populations.

SD = standard deviation.

### 3.3. Medical visits for myopia treatment

A total of 950 patients with myopia sought diagnosis and treatment only in hospitals in 2021. The median direct cost per capita for myopia was 295.00 CNY. In addition, 1292 myopic patients visited ophthalmology clinics, rehabilitation centers, and other nonhospital facilities for diagnosis and treatment. The median annual direct cost per capita for myopia in these settings was 150.00 CNY. Furthermore, within the past year, 557 myopic patients had received treatment at hospitals and other institutions, with a median annual direct cost per capita of 340.00 CNY. The statistical analysis revealed a significant difference in the direct costs associated with myopia (*P* < .01) (Table 3).

**Table 3:** The per capita annual direct cost of myopia for those seeking treatment at hospitals and other medical institutions.

Classification	No.	Mean	SD	Median (*P*25, *P*75)	*P* value*
Only in hospitals	950	402.86	461.45	295.00 (90.00, 500.00)	<.01
Only in other places	1292	334.93	1013.26	150.00 (20.00, 399.00)	
Both in-hospital and out-of-hospital	557	591.97	1106.44	340.00 (122.00, 630.00)	
No myopia-related medical visit	8254	498.61	1471.41	200.00 (40.00, 500.00)	

* Mann–Whitney *U* test for comparing costs of different treatments across correction populations.

SD = standard deviation.

Among the 11,053 individuals diagnosed with myopia, 0.85% underwent myopia surgery, with an average age of 24.09 years. The median cost for preoperative examinations, surgery, eye drops, follow-up, and complications was 6000 CNY, with an average of 10.96 days away from work or school.

### 3.4. The economic burden of myopia

#### 3.4.1. Personal burden of myopia

Myopia is a major public health and economic issue in Yantai, China. Efforts to prevent and treat myopia could have a substantial positive impact on both individuals and society. This study found that the average annual direct cost of myopia per person among 11,053 myopic patients in the past year was 300.00 CNY, and the median cost of seeking medical treatment for various diseases was 500.00 CNY per person, with myopia accounting for 60.00% of the total medical expenses. This finding suggests that myopia imposes a significant economic burden in terms of both direct and indirect costs. The per capita direct cost of myopia was 262.50 CNY in males and 329.00 CNY in females, indicating that the annual direct cost of myopia per capita is higher in females than in males. There was a significant difference in the direct costs of myopia across the different age groups (*P* < .01). Specifically, the highest annual direct cost of myopia was observed in the 6 to 10 years old group at 829.50 CNY, followed by the 11 to 15 years old group at 480.00 CNY. The average annual direct cost of myopia also varied significantly among individuals with different ages of onset (*P* < .01). The direct cost of myopia in children aged 6 to 10 years was 4.7 times higher than that in individuals aged 18 years and above.

There was a significant difference in the direct cost of myopia across different educational levels (*P* < .01). The highest direct cost of myopia was observed among individuals with a primary education or below, at 638.50 CNY. The direct cost of myopia differed significantly among respondents with varying monthly family incomes (*P* < .01). For most Chinese people, family income is private; therefore, this analysis was based on the monthly family income reported by 4892 myopic individuals. The average annual direct cost of myopia was 269.00 CNY for individuals with a monthly income between 20,000 and 49,999 CNY (Table 4).

**Table 4:** The per capita annual direct cost of myopia.

Classification		No.	Mean	SD	Median (*P*25, *P*75)	*P* value*
Gender	Male	5266	661.21	1684.68	262.50 (0.00, 680.00)	<.01
	Female	5787	713.20	1721.77	329.00 (0.00, 750.00)	
Age (yr)	6–10	612	2128.38	4180.69	829.50 (249.00, 2000.00)	<.01
	11–15	3465	1003.40	1975.36	480.00 (200.00, 1000.00)	
	16–25	2754	636.20	1252.72	380.00 (0.00, 760.00)	
	26–35	285	298.81	527.72	0.00 (0.00, 400.00)	
	36–45	3361	258.90	542.58	0.00 (0.00, 399.00)	
	46–55	576	212.57	425.82	0.00 (0.00, 318.42)	
Education level	Primary school and below	1090	1762.58	3697.02	638.50 (200.00, 1686.25)	<.01
	Junior high school	2876	726.29	1578.92	319.00 (319.00, 760.00)	
	High school or secondary vocational school	5018	572.68	1216.20	300.00 (0.00, 670.00)	
	Undergraduate or junior college	1806	345.02	733.65	0.00 (0.00, 498.00)	
	Postgraduate and above	263	389.29	596.24	75.00 (75.00, 590.00)	
Age of myopia onset (yr)	1–6	231	969.42	3645.85	370.00 (0.00, 840.00)	<.01
	7–10	2102	1237.30	2513.49	499.00 (9.50, 1200.00)	
	11–14	4777	711.40	1557.88	380.00 (0.00, 799.00)	
	15–17	2247	419.38	1042.83	150.00 (0.00, 520.00)	
	≥18	1696	261.65	601.22	0.00 (0.00, 360.00)	
Total		11,053	688.43	1704.32	300.00 (0.00, 720.00)	
Household monthly income	<2000 CNY	200	255.21	411.60	0.00 (0.00, 440.00)	<.01
	2000–3999 CNY	592	227.02	475.31	0.00 (0.00, 300.00)	
	4000–5999 CNY	780	240.86	800.35	0.00 (0.00, 360.00)	
	6000–7999 CNY	766	244.56	425.39	0.00 (0.00, 362.59)	
	8000–9999 CNY	1006	240.40	435.47	0.00 (0.00, 360.00)	
	10,000–14999 CNY	813	311.16	671.36	0.00 (0.00, 469.00)	
	15,000–19999 CNY	343	390.51	558.50	200.00 (0.00, 629.00)	
	20,000–49999 CNY	265	572.67	1119.34	269.00 (0.00, 775.00)	
	≥50,000 CNY	127	427.62	585.15	240.00 (0.00, 700.00)	
Total		4892	285.26	619.60	0.00 (0.00, 420.00)	

* Mann–Whitney *U* test for comparing direct cost of myopia across different correction populations.

CNY = Chinese Yuan, SD = standard deviation.

### 3.5. Factors associated with annual direct costs of myopia

To quantify the impact of each predictor on total myopia expenditure, the gamma generalized linear model with a log link was constructed. The direct economic burden per capita of myopia with values >0 is designated as the dependent variable (*y*). The independent variables included gender (0 for male and 1 for female), age at myopia onset, and educational level (1 for primary school, 2 for middle school, 3 for senior high school or technical secondary school, 4 for university or college, and 5 for graduate degree or higher).

The results indicated that age of myopia onset and educational level were significant predictors, whereas gender was not. Age was significantly negatively associated with the direct economic burden (*P* < .01). This indicates that younger individuals incurred significantly higher costs. Specifically, for every 1-year decrease in age, the annual per capita direct cost of myopia increased by approximately 38.53 CNY. Educational level significantly influenced the direct economic burden (with postgraduate and above as the reference group). Patients with primary school education or below incurred an average of 1154.61 CNY more per year (*P* < .01), and those with junior high school education incurred 226.67 CNY more (*P* < .01). No statistically significant differences were observed for high school/vocational school (*P* = .54) or undergraduate/junior college (*P* = .43) compared to the reference group. Overall, a lower educational level was associated with a higher economic burden. Regarding gender, males incurred an additional 39.06 CNY compared to females; however, this difference was not statistically significant (*P* = .39), suggesting that gender is not a significant determinant of the cost amount among those with positive expenditures (Table 5).

**Table 5:** Results of gamma GLM regression analysis for factors associated with total myopia expenditure (N = 7154).

Variable	*B*	SE	*P* value	95% CI
Age at myopia onset	−38.53	3.54	<.01	−45.58 to −31.69
Education level (ref: level = 5)				
Education level = 1	1154.61	145.62	<.01	880.24–1455.77
Education level = 2	226.67	71.96	<.01	89.38–361.02
Education level = 3	40.98	62.58	.54	−83.65 to 157.91
Education level = 4	52.93	68.00	.43	−86.07 to 174.78
Gender (ref: female)				
Male	39.06	46.10	.39	−56.84 to 125.82
Intercept	1324.62	81.95	<.01	1171.79–1491.06

CI = confidence interval, GLM = generalized linear model, SE = standard error.

## 4. Discussion

In this study, the prevalence of myopia among individuals aged 6 to 25 years was found to be 64.78%, which is significantly higher than that reported in Guangzhou’s primary and secondary school students (47.4%)^[9]^ and Han teenagers aged 7 to 18 (57.1%) in the 2014 National College Students Physical Health survey.^[10]^ The highest prevalence of myopia was observed in the 13- to 17-year-old age group, likely because of the heavy academic burden and pressure faced by middle and high school students during critical examination periods. This prolonged period of intense academic work contributes to a notably higher prevalence of myopia compared with other age groups. Our findings suggest that interventions aimed at reducing academic stress and promoting eye health education may be beneficial in mitigating the rising trend of myopia among adolescents.

### 4.1. The younger the age, the heavier the burden of diagnosis and treatment

In recent years, the trend of myopia occurring at younger ages has become a focal point of attention.^[11]^ Studies have indicated that the axial length of the eye reaches adult levels by the age of 14,^[12]^ highlighting the importance of early intervention in controlling myopia development. This study demonstrated that younger age is associated with a heavier burden of correction. Specifically, the per capita direct annual cost of myopia is highest in the 6- to 10-year-old age group, followed by the 11- to 15-year-old myopia group. The 6- to 10-year-old age group represents the initial stage of myopia and is highly valued by parents, resulting in a higher consultation rate and more frequent follow-up visits, which contributes to higher costs. The results of this study are slightly higher than the myopia economic costs reported for middle school students in Kunming^[12]^ but lower than those observed in Beijing.^[13]^ These figures are not adjusted for purchasing power parity; comparisons are indicative, and differences in health-care systems, price levels, and correction patterns limit comparability. Additionally, the per capita direct annual cost of myopia in primary school students and below is 8.51 times that of individuals with a postgraduate degree and above. The age group of 46 to 55 years old exhibits relatively stable vision, with lower frequencies of glass replacement and corresponding costs.

The per capita annual direct cost of myopia varies significantly with the age of onset. Individuals aged 6 to 10 years who developed myopia exhibited a significantly higher per capita annual direct cost of myopia, approximately 3.3 times higher than those who developed myopia at age 15 to 17 years or above. Patients with early-onset myopia are generally at a higher risk of rapid progression of myopia, high myopia prevalence, and complications. Parents of children with myopia often exhibit increased anxiety and concern, which may lead to higher requirements for diagnosis, treatment, and quality of spectacles, potentially increasing overall costs.

### 4.2. Different correction types will affect the economic burden of correction

Among the myopic student population adopting only a single vision correction method, the proportion of those choosing spectacles as the correction regimen is approximately 94.9%. The per capita annual economic burden associated with spectacles in this study was 440.00 CNY, which is comparable to the findings of a study on the economic burden of myopia in Shanghai.^[14]^ Spectacles offer several advantages, including diverse styles, ease of use, and a wide range of options for patients. They typically require replacement every 1 to 2 years and represent the least financial burden on patients with myopia, making them the most acceptable correction method. This result aligns with the findings of Yang Yifang, Yang Linjuan, and others.^[12,15]^ In contrast, contact lens fitting is less convenient, with high cleaning requirements and a high risk of infection. The use of OK/RGP lenses and other corrective methods necessitates regular reviews and follow-up appointments, which must be conducted by professional medical personnel in institutions equipped with the necessary resources. Consequently, these methods incur higher costs. The median cost of spectacles is approximately 1/18 of that of OK/RGP lenses. Additionally, this study observed significant variability in the costs incurred by patients with myopia across different correction methods. This may be attributed to differences in living standards, the variety of spectacles available, prices, and variations in family income.

Studies have shown that the role of wearing spectacles in controlling myopia progression is limited,^[16]^ as evidenced by recent research indicating that the undercorrection of myopia may enhance rather than inhibit its progression.^[17]^ To control the progression of myopia more effectively, some parents of students may choose OK/RGP lenses. However, this intervention could significantly increase the financial burden on rural families, particularly those with limited economic resources.

### 4.3. The location of spectacle fitting and the frequency of replacement affect the economic burden of myopia

The per capita annual direct cost of myopia is the highest among individuals who sought treatment exclusively both in and out of the hospital within the past year. This disparity could be attributed to several factors. First, patients opting for hospital-based care are more inclined to select costly correction methods, such as OK/RGP lenses or refractive surgery, which significantly contributes to the economic burden. Second, individuals receiving treatment at specialized institutions may exhibit higher expectations regarding the quality of spectacles, thereby increasing costs, and the frequency of eyeglass replacement plays a notable role in the economic impact of myopia, leading to increased expenses.^[12]^

### 4.4. Economic burden

The results showed that the per capita direct cost of myopia in Yantai was 300.00 CNY/yr. According to data released by the National Bureau of Statistics, the average annual salary of non-private sector employees in urban areas in China was 106,837 CNY in 2021, while the average annual salary of private sector employees was 62,884 CNY. The average annual salary of non-private sector employees in urban areas of Shandong province was 94,768 CNY, and the average annual salary of non-private sector employees in Yantai was 94,524 CNY. Based on a conservative estimate using the average annual salary of private-sector employees in China (62,884 CNY), myopia-related annual expenditures constitute approximately 0.48% of their total income. Similarly, employing a rough estimate based on the average annual salary of non-private sector employees in Yantai (94,524 CNY), myopia-related annual expenditures account for roughly 0.32% of their income.

The significant negative association between age and direct economic burden indicates that younger patients incurred substantially higher annual costs. Each 1-year decrease in age was associated with an increase of approximately 38.53 CNY in annual per capita direct expenditure. This gradient is consistent with recent evidence from a systematic literature review, which reported that healthcare utilization for myopia is highest among children aged 5 to 9 years and declines with age in China.^[14]^ Furthermore, early-onset myopia carries a higher risk of rapid progression and subsequent high myopia, prompting more intensive management strategies such as OK/RGP lens or low-dose atropine, which substantially increase expenditures.

Educational attainment demonstrated an inverse gradient with direct economic burden. Compared with patients with postgraduate education or above, those with primary school education or below incurred an additional 1154.61 CNY/yr, and those with junior high school education incurred an additional 226.67 CNY. High school/vocational school and undergraduate/junior college groups did not differ significantly from the reference group. These results align with the broader observation that the economic burden associated with myopia management is largely driven by vision correction procedures and specialist visits, with notable socioeconomic disparities across regions.^[18,19]^ Since the educational level in our study population is closely tied to age and school stage, the higher costs observed in lower educational groups likely reflect the combined effects of younger age, earlier myopia onset, and the associated parental investment patterns described above. It is also plausible that families of younger children are more likely to seek care at specialized institutions and opt for more expensive correction methods, thereby driving up direct costs. These findings suggest that the economic burden of myopia in Yantai is concentrated among the youngest patients and those at earlier educational stages, who are also most likely to be in the critical window for myopia progression.

This study has limitations regarding the cost-of-illness of myopia in Yantai. First, the potential for recall bias when participants complete questionnaires may affect the accuracy of cost-related data; therefore, the study results represent a conservative estimate of the true burden. Second, patient-reported myopia severity and related data may have introduced bias. Third, as a cross-sectional study without projection modeling or discounting future costs, our estimates describe the current burden rather than projecting future costs. Additionally, extrapolation to other regions or to the national level should be made with caution because of differences in demography, health-care prices, and correction practices. Future research should focus on calculating both the direct and indirect costs associated with myopia and employ more robust methods to minimize biases. The effectiveness of existing interventions should be evaluated to provide scientific evidence for the development of effective intervention policies. This study did not account for indirect costs related to myopia, which may limit the comprehensiveness of the cost-of-illness. Future studies should continue to explore various aspects of the social and economic burden of myopia and determine the specific factors that cause this burden and their effects on specific populations.

Myopia is a significant public health concern, and its associated economic burden is substantial, particularly in regions such as Yantai, where the prevalence of myopia is high. The multifaceted nature of the social and economic burden related to the diagnosis and treatment of myopia underscores the complexity of this issue. Given the ongoing increase in myopia prevalence rates, it is imperative to systematically evaluate the social and economic impacts of myopia. Such assessments can provide quantitative insight into the broader economic and social consequences of this condition. In response, governments must implement effective interventions aimed at reducing both the prevalence and progression of myopia while ensuring equitable access to diagnostic and therapeutic services for all populations. Furthermore, future research should continue to delve into various dimensions of the socioeconomic burden of myopia, including its specific effects on different demographic groups as well as the potential roles of innovative interventions and technologies in mitigating this condition.

## 5. Conclusion

The economic burden of myopia is higher among younger age groups, those with early-onset myopia, and those who opt for more expensive correction methods. The location of eyeglass fitting and the frequency of replacement also significantly influence the economic burden of myopia. These findings suggest that promoting myopia prevention and control is crucial for reducing the economic burden. Enhancing public awareness of myopia prevention strategies and encouraging the use of cost-effective correction methods are essential steps to address this issue.

## Author contributions

**Conceptualization:** Yuelin Sun.

**Data curation:** Yuelin Sun, Xiaosong Cheng, Cong Sun, Guiqiang Wang.

**Formal analysis:** Fei Wang, Guiqiang Wang.

**Funding acquisition:** Yuelin Sun, Guiqiang Wang.

**Investigation:** Xiaosong Cheng, Cong Sun, Naibao Hu.

**Methodology:** Xiaosong Cheng, Cong Sun, Xiaoyue Wang.

**Project administration:** Xiaoxiang Jin.

**Resources:** Yuelin Sun, Xiaosong Cheng.

**Software:** Yuelin Sun, Naibao Hu, Xiaoxiang Jin, Xiaoyue Wang.

**Supervision:** Yuelin Sun, Guiqiang Wang.

**Validation:** Yuelin Sun.

**Visualization:** Yuelin Sun.

**Writing – original draft:** Yuelin Sun, Guiqiang Wang.

**Writing – review & editing:** Yuelin Sun.
